# TUMORS OF THE PATELLA: THE EXPERIENCE OF INSTITUTE OF ORTHOPEDICS AND TRAUMATOLOGY AT UNIVERSITY OF SÃO PAULO, BRAZIL

**DOI:** 10.1590/1413-785220162403159158

**Published:** 2016

**Authors:** ANDRÉ MATHIAS BAPTISTA, SYLVIO CESAR SARGENTINI, JUAN PABLO ZUMÁRRAGA, ANDRÉ FERRARI DE FRANÇA CAMARGO, OLAVO PIRES DE CAMARGO

**Affiliations:** 1. Universidade de São Paulo, Faculdade de Medicina, Hospital das Clínicas, Instituto de Ortopedia e Traumatologia, São Paulo, SP, Brazil.; 2. Instituto do Câncer do Estado de São Paulo, Grupo de Oncologia Ortopédica, São Paulo, SP, Brazil.; 3. Universidade de São Paulo, Faculdade de Medicina, Department of Orthopedics and Traumatology, São Paulo, SP, Brazil.

**Keywords:** Patella, Bone neoplasms, Biopsy.

## Abstract

**Objective:**

To obtain epidemiological data from the tumors of the patella diagnosed and treated at the Instituto de Ortopedia e Traumatologia do Hospital das Clínicas da Universidade de São Paulo (IOT-HC-FMUSP) between 1998 and 2015.

**Methods:**

Series of cases with retrospective evaluation of patients diagnosed with tumors located in the patella. The data was obtained from the records and patients' charts at the Department of Pathology of IOT-HC-FMUSP.

**Results:**

A total of 2220 medical records from patients with anatomopathological reports were included in the study. Only eight (0.3%) patients had patellar tumors. We found that six (75%) of these were benign, one (12.5%) was a pseudotumoral lesions and one (12.5%) was reported as malignant. Among benign tumors, the giant cell tumor (GCT) was the most frequently reported corresponding to 50% of the cases. Hemagioendothelioma was the only case of malignant tumor in this series. As for the pseudotumoral lesions, we found a brown tumor.

**Conclusion:**

From the data obtained retrospectively in a 17 year time frame, in a service that treats benign, malignant and pseudotumoral bone lesions, we conclude that our casuistry in patellar tumors is similar to that reported in scientific literature, where benign tumors are predominant in a 7:1 ratio over malignant tumors, being a rare location of appearance, with the GCT as the most common diagnosis *. Level of Evidence IV, Case Series.*

## INTRODUCTION

The patella is an infrequent location for the onset of benign or malignant bone tumors, as well as pseudotumoral lesions. There are few published studies on this topic, and they are mostly case series. They present clinically with severe pain in the anterior knee or an obvious growth of soft tissue. A large number of differential diagnoses should be considered when evaluating the appearance of tumors in the patella.^1^ Tumors in the patella represent 0.12% of all bone tumors and the lesions are mostly benign. The most common tumor reported in the literature is giant cell tumor (GCT), followed by chondroblastoma. Among malignant tumors, the most common is the hemangioendothelioma, followed by lymphoma and osteosarcoma (OS).^1^ ^-^ ^3^

Although the tumors are usually benign, they require special attention, since the patella plays an irreplaceable biomechanical function in knee extension, increasing the lever arm of the quadriceps muscle of the thigh, providing an increase of up to 50% in extensor strength. With this action, the patella brings 60% more torque for the last 15° for total knee extension.^4^ ^,^ ^5^ The main purpose of this study was to present epidemiological data of patellar tumors at our service.

## MATERIALS AND METHODS

The study was approved by the Ethics Committee of the Department of Orthopedics and Traumatology, IOT-HCFMUSP, under number 8 319/2012. This is a retrospective study where we used the medical records of patients diagnosed with patellar tumors treated at the Orthopedic Oncology Group of IOT-HC-FMUSP, from 1998 until early March 2015.

We studied 2.220 anatomopathological results of patients who underwent a biopsy in this 17 year period. Eight (0.3%) patients with tumor on the patella were included. We obtained from medical records the following epidemiological information: age, gender, main complaint, and histological diagnosis. We also used the anteroposterior, lateral and axial patellar radiographs to locate the exact site of the disease, as well as its characteristics.

Regarding the study sample, the mean age of patients was 24 ± 12 years old. Five (57.15%) patients were male and three (42.85%) were female. The most common location on the patella was the upper pole, with three cases (37.50%), followed by the body, with three cases (37.50%) and finally the inferior pole with two cases (25%). Data were expressed as mean and standard deviation.

### STATISTICAL ANALYSIS

Nominal characteristics of patients were described by relative absolute frequencies. The ages of the patients were described as mean and standard deviation.^6^

The type of tumor and its variations in the location on the patella were described with summary measures (mean, standard deviation, median, minimum and maximum). Correlations were calculated between anatomopathological diagnosis, radiographic characteristics and condition of the knee joint with Spearman's correlation coefficient to verify the correlation between them. The tests were performed at 5% significance level. The software used for the statistical analysis was SPSS 20.0 (Statistical Program for Social Sciences for Windows version 20.0) and Microsoft Excel 2008.

## RESULTS

Regarding the clinical presentation, seven patients (87.5%) had only knee pain and one patient (12.5%) showed increased local volume associated with decreased strength for knee extension. Radiographically, seven cases (87.5%) presented as lytic injuries and one case (12.5%) presented as sclerotic lesion. We found no involvement of the articular surface.

The most frequent anatomopathological diagnosis was the GCT with three cases (37.5%), followed by one of each of the following cases: aneurysmal bone cyst (ABC), osteoid osteoma, chondroblastoma, hyperparathyroidism brown tumor, and hemangioendothelioma, representing each one 12.5%.

**Figure 1:**
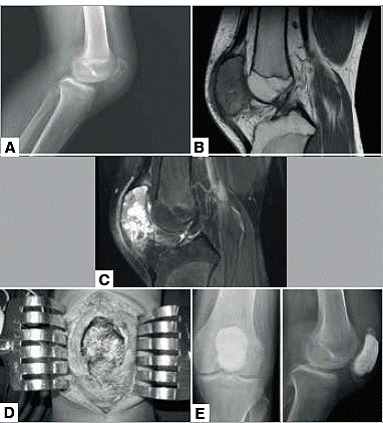
Aneurysmal bone cyst (case 8). (A) Profile x-ray of the knee; (B) Sagittal NMR T1; (C) Sagittal NMR T2; (D) Intraoperative; (E) AP+P/Postoperative x-ray.

Of eight patients, five (62.5%) were treated with curettage and three (37.5%) with partial patellectomy. The inferior pole was resected in two cases (66.6%) and one (33.3%) resection of the lateral facet. Of the five cases treated with curettage, three (60%) received polymethylmethacrylate (PMMA), one (20%) received autologous bone graft and one (20%) remained unfilled. (Table 1 and Figure 1A-F)

**Table 1:** Case distribution by chronological order.

Cases	Year	Gender	Age (years old)	Diagnosis	Treatment	MSTS
Case 1	1998	M	17	Hemangioendothelioma	Partial patellectomy	#
Case 2	1999	F	31	GCT	Curettage + PMMA	100%
Case 3	1999	F	26	GCT	Partial patellectomy	90%
Case 4	2002	M	17	Chondroblastoma	Curettage + PMMA	90%
Case 5	2003	M	25	GCT	Partial patellectomy	100%
Case 6	2009	F	41	Brown tumor	Curettage + PMMA	100%
Case 7	2011	M	12	Osteoid osteoma	Partial patellectomy	100%
Case 8	2013	M	19	ABC	Curettage + PMMA	80%

All patients had good functional outcomes, measured by the functional score of the Musculoskeletal Tumor Society (MSTS), with an average score of 93.8%. Patients treated with partial patellectomy had an average score 93.3% and those treated with intralesional resection had on average 94% in the MSTS functional score.

There were no cases of recurrence. There has been one surgical complication for the patient treated with curettage and PMMA, who obtained a functional score of 80%. The main complaint was pain and knee flexion difficulty due to extravasation of PMMA in the joint. We performed a surgical rapprochement for PMMA regularization and interposition of intraarticular fascia lata.

## DISCUSSION

The patella is a sesamoid bone where the appearance of bone tumors is extremely atypical. Due to its importance for normal knee function, specifically in the extensor mechanism, performing a correct and early diagnosis is important. Besides being a rare place of presentation, we found no predilection for a specific type of tumor, which is why a large number of differential diagnoses should be considered. Dahlin,^7^ in a study with 7.975 patients with bone tumors, showed that only ten cases were located in the patella. Among the diagnosis, six were benign tumors, the most common being chondroblastoma. Four cases were malignant tumors, hemangioendothelioma being the most frequent. In a similar study, Ehara et al.^8^ published 23 cases of patellar tumors following the same pattern, chondroblastoma being the most common benign tumor. The main difference between this study and others is that most reported injuries were pseudotumoral ones.

In the present study, among 2.220 cases, eight were located in the patella. We also observed that most of them were benign, the most common being the GCT. The pseudotumoral lesions and malignant tumors are equally frequent, with one case each. Similarly to the literature, hemangioendothelioma was the reported malignant tumor.

In a study published by Bhagat et al.,^9^ seven patients with tumors in the patella were evaluated. Most were male with a mean age of 42.5 years old with significant pain complaints in the anterior knee. They showed no malignant tumors in this study. From the diagnoses presented, the most common was the GCT followed by chondroblastoma, two of them with pathological fracture. The treatment option was always surgical, having as first option curettage with autograft for chondroblastoma and full patellectomy in cases of GCT.

Our study also showed males as the most affected by patellar tumors. The mean age varies widely, ours was 24 ± 12 years old. All our cases showed pain in the anterior knee as a clinical symptoms and we did not find pathological fractures. As treatment option we also always performed surgery with curettage and partial patellectomies. 

Due to the fact that the patella is an unusual location for bone tumors, most studies found in the literature are case series. Vallianatos et al. ^10^ and Franceschi et al. ^11^ reported cases of osteoid osteoma in the patella treated by CT scan guided ablation. De Coster et al. ^12^ described a case of osteoblastoma the patella in a 29 year old patient. The authors reported that there is no description in the literature of this type of tumor in the patella. Similarly, Yoshida et al. ^13^ described GCT of the patella as a rare case, however, in this study the authors conducted a literature search together with the Orthopedics Japanese Association on GCT diagnostic, finding that since 1972 there were only 22 tumors located in the patella. (Table 2)^1^ ^,^ ^3^ ^,^ ^7^ ^-^ ^9^ ^,^ ^14^ ^,^ ^15^

**Table 2:** Case series by chronological order

Article	Total number of patients (n)	Mean age (years old)	M / F	Benign	Malignant	Pseudotumors
Cole et al. (1925)^3^	21	27.6	11 (52.38%) / 10 47.62%)	7 (33%)	13 (61%)	1 (6%)
Dahlin et al. (1968)^7^	10	#	#	6 (60%)	4 (40%)	0 (0%)
Ehara et al. (1989)^8^	23	#	#	5 (21.74%)	4 (17.40%)	14 (60.81%)
Ferguson et al. (1997)^14^	8	#	#	6 (75%)	2 (25%)	0 (0%)
Saglik et al. (2008)^15^	8	#	#	5 (62.5%)	2 (25%)	1 (12.5%)
Bhagat et al. (2008)^9^	7	42.5	5 (71.43%) / 2 (28.57%)	5 (71.43%)	0 (0%)	2 (28.58%)
Singh et al. (2009)^1^	59	32	42 (71.2%) / 17 (28.8%)	23 (39%)	9 (15.25%)	27 (45.75%)

Finally, we believe that this study describes our experience with tumors in the patella, in accordance with the literature, confirming the lack of information and publications on this topic.

## CONCLUSION 

From the data obtained in a 17 year retrospective survey in a service that reports and treats benign, malignant, and pseudotumoral bone lesions. We found that our sample of patellar tumors proves to be consistent with the literature on the prevalence ratio of benign to malignant tumors of 7: 1. Tumors in the patellar are a rare presentation place, where the GCT is the most common diagnosis.

## References

[1] Singh J, James SL, Kroon HM, Woertler K, Anderson SE, Jundt G (2009). Tumour and tumour-like lesions of the patella--a multicentre experience. Eur Radiol.

[2] Mercuri M, Casadei R (2001). Patellar tumors. Clin Orthop Relat Res.

[3] Cole WH (1925). Primary tumors of the patella. J Bone Joint Surg.

[4] Kaufer H (1971). Mechanical function of the patella. J Bone Joint Surg Am.

[5] Lieb FJ, Perry J (1968). Quadriceps function An anatomical and mechanical study using amputated limbs. J Bone Joint Surg Am.

[6] Kirkwood BR, Sterne JA (2006). Essential medical statistics.

[7] Dahlin DC (1968). Tumors of the patella. JAMA.

[8] Ehara S, Khurana JS, Kattapuram SV, Rosenberg AE, el-Khoury GY, Rosenthal DI (1989). Osteolytic lesions of the patella. AJR Am J Roentgenol.

[9] Bhagat S, Sharma H, Bansal M, Reid R (2008). Presentation and outcome of primary tumors of the patella. J Knee Surg.

[10] Vallianatos PG, Tilentzoglou AC, Seitaridis SV, Mahera HJ (2006). Osteoid osteoma of the patella a case report. Knee Surg Sports Traumatol Arthrosc.

[11] Franceschi F, Longo UG, Ruzzini L, Marinozzi A, Rizzello G, Papalia R (2008). En-bloc retrograde resection of an osteoid osteoma of the patella using computed tomography under arthroscopic control. J Knee Surg.

[12] De Coster E, Van Tiggelen R, Shahabpour M, Charels K, Osteaux M, Opdecam P (1989). Osteoblastoma of the patella Case report and review of the literature. Clin Orthop Relat Res.

[13] Yoshida Y, Kojima T, Taniguchi M, Osaka S, Tokuhashi Y (2012). Giant-cell tumor of the patella. Acta Med Okayama.

[14] Ferguson PC, Griffin AM, Bell RS (1997). Primary patellar tumors. Clin Orthop Relat Res.

[15] Saglik Y, Yildiz Y, Basarir K, Tezen E, Güner D (2008). Tumours and tumour-like lesions of the patella a report of eight cases. Acta Orthop Belg.

